# Integrating Genomics, Radiomics, and Pathomics in Oncology: A Scoping Review and a Framework for AI-Enabled Surgomics

**DOI:** 10.3390/bioengineering13010117

**Published:** 2026-01-20

**Authors:** Selma Mtoor, Niki Rashidian, Nouredin Messaoudi, Vincent Grasso, Floriane Noel, Michele Steindler, Derar Jaradat, Isabella Frigerio, Giovanni Butturini, Roland Croner, Karol Rawicz-Pruszynski, Giulia Capelli, Gaya Spolverato, Marc G. Besselink, Takeaki Ishizawa, Elie Chouillard, Mohammad Abu-Hilal, Ulf Kahlert, Ibrahim Dagher, Andrew A. Gumbs

**Affiliations:** 1 Department of Biomedical, University Duisburg-Essen, 45141 Essen, Germany; selmamtoor@gmail.com; 2 Department of General, HPB Surgery and Liver Transplantation, Ghent University Hospital, Corneel Heymanslaan 10, 9000 Ghent, Belgium; niki.rashidian@gmail.com; 3 Ziekenhuis Brussel (UZ Brussel) and Europe Hospitals, Laarbeeklaan 101, 1090 Brussels, Belgium; nouredinmessaoudi@gmail.com; 4 Department of Electrical and Computer Engineering, University of New Mexico, Albuquerque, NM 87106, USA; vincent.grasso@gmail.com; 5 Department of Medical Education and Scholarship, Rowan-Virtua School of Osteopathic Medicine, Rowan University, Stratford, NJ 08084, USA; 6 Department of Surgical Oncology, Medical University of Lublin, Radziwiłłowska 13 St., 20-080 Lublin, Poland; karol.rawicz-pruszynski@umlub.edu.pl; 7 Smart4 Tech Engineering, Sibylone, 75002 Paris, France; fnoel@sibylone.com (F.N.); michele.steindler@gmail.com (M.S.); 8 Department of Surgery, Medical University of Graz, 8010 Graz, Austria; 9 Department of Surgery, University of Essen, 45141 Essen, Germany; 10 Department Surgery, HPB Unit, Pederzoli Hospital, Peschiera del Garda, 37019 Verona, Italy; 11 Collegium Medicum, University of Social Sciences, 90-237 Łodz, Poland; 12 Department of General-, Visceral-, Vascular- and Transplantation Surgery, University of Magdeburg, 39120 Magdeburg, Germany; roland.croner@med.ovgu.de (R.C.);; 13 Department of Surgery, ASST Bergamo Est, Seriate, 24068 Bergamo, Italy; 14 Department of Surgical, Oncological and Gastroenterological Sciences (DiSCOG), University of Padova, 35121 Padova, Italy; gaya.spolverato@gmail.com; 15 Department of Surgery, Amsterdam Gastroenterology Endocrinology Metabolism, Amsterdam UMC, University of Amsterdam, 1105 Amsterdam, The Netherlands; 16 Department of Hepato-Biliary-Pancreatic Surgery, Graduate School of Medicine, Osaka Metropolitan University, Osaka 545-8585, Japan; take1438@gmail.com; 17 American Hospital of Paris, 55 Bd du Château, 92200 Neuilly-sur-Seine, France; 18 Department of Surgery, School of Medicine, The University of Jordan, Amman 11942, Jordan; 19 Department of Surgery, University Hospital Southampton NHS Foundation Trust, Southhampton SO16 6YD, UK; 20 Hôpital Antoine Béclère, Assistance Publique-Hôpitaux de Paris, 157 Rue de la Porte de Trivaux, 92140 Clamart, France

**Keywords:** precision medicine, genomics, radiomics, pathomics, surgomics, personalized medicine

## Abstract

Background: Multimodal AI integration across genomics, radiomics, and pathomics is rapidly evolving in oncology, but evidence remains heterogeneous and unevenly distributed across modalities. Objective: To map empirical studies integrating two or more -omic modalities, summarize integration and validation approaches, and identify gaps informing future directions toward surgomics. Methods: We conducted a scoping review in accordance with PRISMA-ScR, searching PubMed, Ovid, Wiley Online Library, and Google Scholar for English-language studies published from January 2020 to 5 March 2025. We charted study characteristics, modalities combined, fusion strategies, AI model categories, validation approaches, and reported performance metrics as presented by the original studies. Results: From 184 records, 11 studies met inclusion criteria (*n* = 1078 total participants across reported studies), most focusing on radiomics–pathomics integration; fewer incorporated genomics, and tri-modal fusion was uncommon. Studies varied widely in clinical tasks, endpoints, preprocessing, and validation, limiting direct comparability. Conclusions: The mapped evidence indicates growing methodological activity in radiopathomics and cross-scale association modeling, while tri-modal pipelines and clinically deployable multimodal workflows remain underdeveloped. Surgomics is presented as a conceptual, staged roadmap informed by these gaps rather than a current clinical capability.

## 1. Introduction

**Figure 1:**
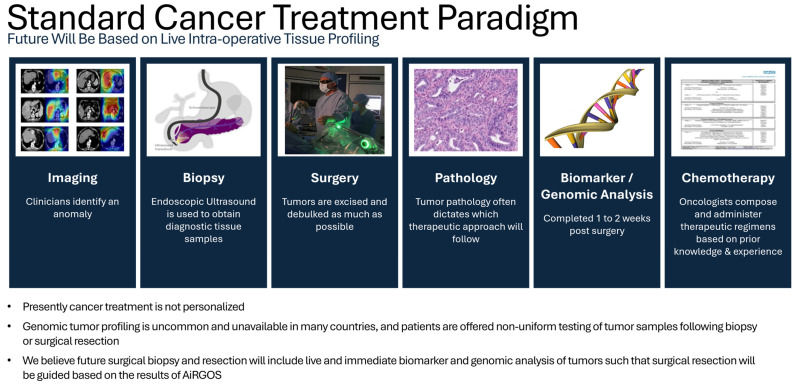
Standard Cancer Treatment Paradigm. Imaging (open access) courtesy of Professor Takeaki Ishizawa’s team [11]. **DNA double helix (public domain).** Source: National Institutes of Health (NIH), via Wikimedia Commons. https://commons.wikimedia.org/wiki/File:DNA_Double_Helix.png (accessed on 23 November 2025).

Currently, tumor boards only use a fraction of the data available when making treatment decisions for patients with cancer (Figure 1). Precision medicine has transformed the field of healthcare by providing customized therapies that are based on an individual’s specific traits [1]. The practice of precision medicine necessitates the capacity to categorize patients into distinct groups that vary in their vulnerability to a certain illness, the biological characteristics of the illness, and their reaction to treatment [2]. Genomics, radiomics, and pathomics are important factors that contribute to the advancement of precision medicine. These fields allow for a more comprehensive knowledge of illnesses at the molecular, imaging, and tissue pathology levels, respectively [3,4,5]. Although all eleven studies met the multi-omics inclusion criteria, the depth of integration varied. Comparative results indicate that 8 of 11 studies reported measurable gains in predictive or diagnostic performance versus single-omics baselines. These gains, while encouraging, are limited by small sample sizes and inconsistent validation strategies.

**Figure 2:**
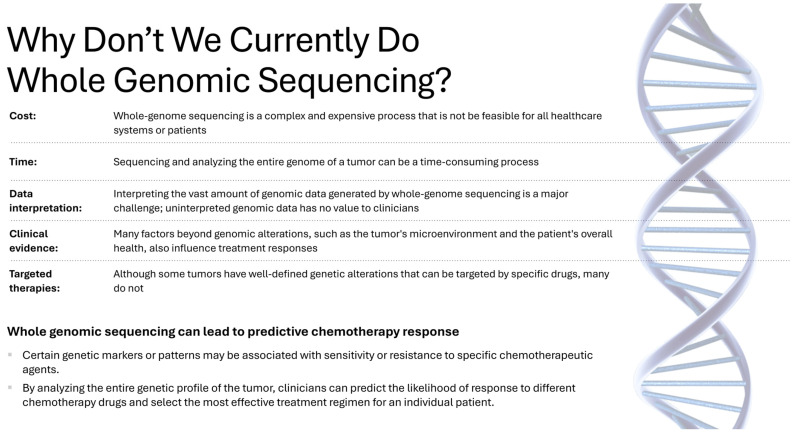
Why don’t we currently do Whole Genomic Sequencing?

Genomics has been instrumental in advancing our understanding of genetic variations and their links to different health conditions. Initiatives such as the Human Genome Project have made significant strides in identifying somatic point mutations, copy number alterations, translocations, and gene fusions. This research provides valuable clinical information that is relevant to patient care [6]. Although sequencing costs have decreased, routine clinical integration of WGS into multimodal AI pipelines remains constrained by turnaround time, sample logistics, analytical workflows, and governance requirements; therefore, most current clinical implementations rely on targeted panels or whole-exome sequencing rather than intraoperative WGS (Figure 2). A research study conducted by Jung et al. [7] used whole-exome sequencing to investigate genetic alterations in pulmonary sclerosing pneumocytoma. The investigation demonstrated a significant occurrence of AKT1 point mutations, with 31 out of 68 individuals (46%) affected, including the p.E17K variant. AKT1 mutations have been proposed as the genetic characteristic of pulmonary sclerosing pneumocytoma [8]. A recent investigation showed that the mTOR pathway is consistently genetically altered in pulmonary sclerosing pneumocytoma [9]. The PI3K/AKT/mTOR pathway is a commonly active carcinogenic pathway [10]. AKT, when activated, phosphorylates mTOR, leading to the activation of mTORC1.

Radiomics is a cutting-edge technique for analyzing medical images. It uses data mining and machine learning (ML) to extract numerous complex features that are not visible to the human eye. These features are then used in a clinical decision support system [12,13]. Radiomics is very valuable in the characterization of diseases, staging tumors, evaluating the effectiveness of treatments, and predicting prognosis [3,13]. Recent studies in the field of glioma have shown promising findings in establishing connections between magnetic resonance imaging (MRI) characteristics and differential diagnosis [14], molecular characteristics [15,16], and prognoses [17,18,19].

Pathomics is a novel technique that integrates pathology, imaging, and computer science to comprehend disease processes. Pathomics has transformed the detection and treatment of illnesses by digitizing and automating the interpretation of histological images via the use of computer vision and ML techniques. Computers have the ability to recognize and extract items, such as cells and blood arteries, from digital pictures. They can also categorize and describe these entities. This data enhances the categorization, assessment of severity, predictions of outcomes, and development of treatment strategies for diseases. Pathomics has the capacity to uncover the configuration and organization of tumor cells and the surrounding tissue milieu [20,21]. The whole slide images (WSI) provide a comprehensive representation of several physical traits of cancer and the danger it poses to patients in many organs [22], such as the lungs [23]. Various pathomics strategies, such as topological [24], morphological [25], and convolutional neural networks (CNNs) strategies, have been employed to analyze lung cancer specimens on digitized slides stained with hematoxylin and eosin (H&E) [26]. These strategies aim to identify and understand the intricate patterns that distinguish different pathological subtypes of lung cancer. Given that computed tomography (CT) and digital pathology provide distinct but complementary information on the tumor in vivo, it is worth exploring the potential connection between radiomic and pathomic characteristics. The possible combination of genomics, radiomics, and pathomics has the potential to greatly transform healthcare and enhance patient outcomes.

Surgomics is a developing field of healthcare that integrates data involving the preoperative, intraoperative, and postoperative care of the surgical patient. Similarly to other -omics, it combines information gleaned from radiomics, pathomics, transcriptomics, and proteomics (e.g., mult-iomics) in the preoperative and postoperative phase but also integrates intraoperative video data during the intraoperative phase to generate useful information via ML algorithms and deep learning (DL) architectures that can, in theory, enable surgeons to make more evidence-based clinical decisions. It has been divided into integrated surgomics and video surgomics to highlight the potential for intraoperative video to provide further data points for enhanced AI analysis. Integrated surgomics involves the preoperative and postoperative evaluation of every data point (e.g., genomics, radiomics, pathomics) involved in the care of surgical patients. Video surgomics involves the analysis of intraoperative video data with preoperative multi-omic data. In the long term, and subject to major workflow, regulatory, and technical constraints, surgomics could support intraoperative decision support; in the near term, the most realistic impact is pre-/post-operative multimodal decision support. For example, it would be enormously helpful for surgeons to know which patients with cancer would benefit from a radical resection and which ones would not. It is hoped that surgomics will be able to finally enable truly personalized care even in the operating room.

This Scoping Review first synthesizes evidence from 11 studies to evaluate how integrating more than 2-omics layers (e.g., radiomics-pathomics) improves diagnostic and predictive accuracy in cancer care. Beyond synthesizing these findings, we identify a critical gap: no studies yet combine genomics, radiomics, pathomics, and intraoperative data. This motivates our proposal of surgomics—a framework to unify multi-omics with AI-driven intraoperative video analytics. By bridging this gap, surgomics could enable real-time decision support, transforming precision surgery. This review differs from prior narrative reviews by (i) systematically synthesizing post-2024 empirical evidence, (ii) summarizing integration strategies, validation practices, and performance reporting patterns across studies, and (iii) proposing “surgomics” as a forward-looking roadmap rather than a current clinical capability. We explicitly separate the evidence synthesis from the conceptual framework to avoid conflating present evidence with future directions. Accordingly, this scoping review summarizes how integration is performed (fusion strategies and AI methods), characterizes validation practices, and identifies gaps that motivate future research directions. A separate, clearly delineated section presents surgomics as a conceptual roadmap informed by (but distinct from) the mapped evidence.

## 2. Methods

### 2.1. Search Strategy

A comprehensive literature search was conducted across PubMed, Ovid, Wiley Online Library, and Google Scholar from 1 January 2020–5 March 2025. The review protocol was prospectively registered in PROSPERO (CRD420251009238) on 11 March 2025, prior to data extraction and synthesis. We selected 2020 onward to capture contemporary AI-enabled multimodal integration studies while retaining sufficient empirical volume for scoping review synthesis. Seminal pre-2020 works were cited only for background context and were not included in the evidence synthesis. However, to provide appropriate background context, seminal multi-omics studies published before 2020 were considered in the Introduction and Discussion but not included in the synthesis, as they did not meet the inclusion criteria for temporal scope or data completeness.

This scoping review was conducted in accordance with the PRISMA-ScR guidelines. The objective was to map the scope of empirical evidence on multimodal integration of genomics, radiomics, and pathomics in oncology, summarize methodological approaches (integration strategies, AI model categories, and validation practices), and identify evidence gaps relevant to future multimodal and surgomics development. This Scoping Review protocol was registered on PROSPERO on 11 March 2025, registration number CRD420251009238. A comprehensive search strategy was devised by integrating MeSH terms and relevant keywords from the literature. The search criteria included the terms “Precision medicine”, “personalized medicine”, or “stratified medicine” along with “genomics” or “gene*” and “radiomics” or “medical images” and “pathomics”. Full search syntax for all databases. A cross-referencing process was used to identify other relevant research within the reference lists of the included publications. This research adhered to the PRISMA guidelines for its execution [27]. The scientists made individual decisions on the inclusion of titles and abstracts. A rigorous assessment was conducted to evaluate the potential for bias in the papers that were included in the analysis, namely in the field of medical and health sciences. The protocol was finalized before screening and data extraction. The database searches were executed up to 5 March 2025, and the protocol was registered in PROSPERO on 11 March 2025, prior to full-text screening, data extraction, and quality appraisal, thereby minimizing selective reporting risk. The protocol was registered in PROSPERO on 11 March 2025. Registration occurred after preliminary piloting of eligibility criteria but before final data charting and synthesis; the scoping aims remained exploratory and focused on evidence mapping rather than quantitative effect estimation.

### 2.2. Screening and Selection Workflow

Two independent reviewers performed the initial title and abstract screening, followed by full-text assessment of potentially eligible studies. Any disagreements at either stage were resolved through discussion and consensus with a third senior reviewer. Inter-reviewer agreement for study selection was quantified using Cohen’s κ statistic, which demonstrated substantial agreement (κ = 0.82), registered on 11 March 2025, prior to data extraction and synthesis. All records were managed using Rayyan (version 1.6) to facilitate blinded screening and duplicate removal. The use of Rayyan also allowed automatic detection of overlapping records and tagging of inclusion/exclusion reasons. After consensus resolution, the final list of included studies was exported for data extraction and quality appraisal.

### 2.3. Inclusion and Exclusion Criteria

Eligibility criteria included English-language studies published between 1 January 2020–5 March 2025, that reported empirical integration of ≥2 modalities (genomics, radiomics, pathomics) with quantitative evaluation, written in English, and specifically focused on genomics and radiomics; genomics and pathomics; pathomics and radiomics; and genomics, pathomics, and radiomics in the context of healthcare. The eligibility criteria included peer-reviewed original research articles, case reports, and validated preprints that presented primary data on the integration of ≥2 omics layers (genomics, radiomics, pathomics) in clinical or experimental settings. Conceptual frameworks or methodological proposals were only included if they contained empirical findings relevant to multi-omics integration or surgomics development. Case reports were included only for descriptive synthesis, providing illustrative examples of multi-omics application in unique clinical contexts. They were not incorporated into the quantitative or pooled narrative synthesis to avoid inflating generalizable conclusions. Review articles, editorials, conference proceedings without full peer review, and conceptual proposals lacking primary data were excluded. The included studies primarily employed machine-learning pipelines such as handcrafted radiomics feature extraction (PyRadiomics) with XGBoost classifiers, multiple-instance learning (MIL) on whole-slide images, convolutional neural network (CNN) fusion with clinical features, and deep orthogonal fusion (DOF) architectures. No eligible radiogenomic studies were included, as none within the 2020–2025 search window met the predefined inclusion criteria (peer-reviewed, human data, multimodal integration). Several were excluded for incomplete validation or missing performance metrics.

**Figure 3:**
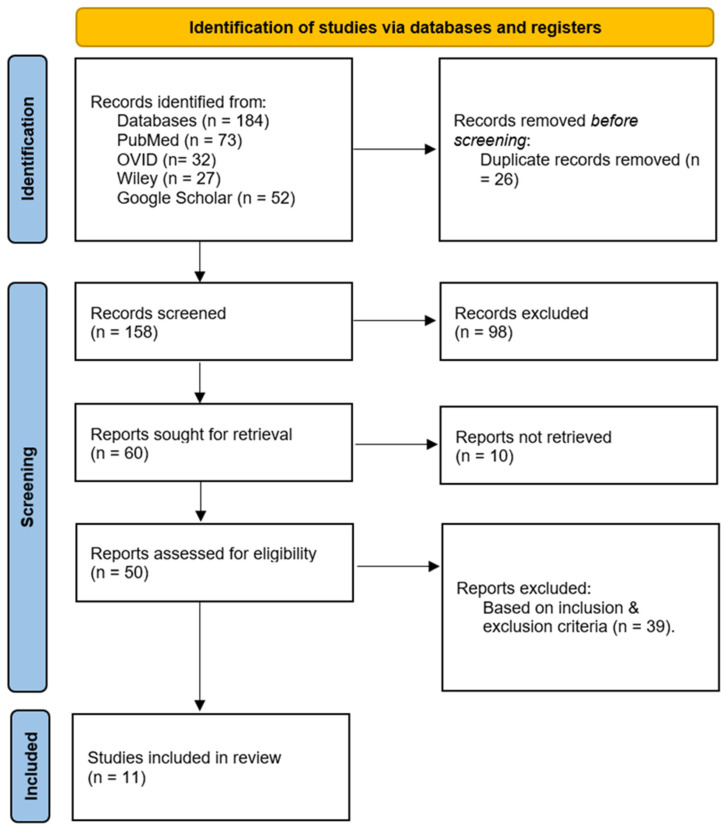
PRISMA-ScR flow diagram.

“Integrated” was defined as explicit multimodal fusion at the feature level or model level (e.g., joint feature concatenation, multimodal encoders with late fusion, or unified training across modalities). Studies that only reported separate unimodal analyses without fusion were not considered integrated. “Quantitative outcomes” were defined as reported numerical performance or clinical metrics, including AUC, C-index, specificity, calibration, decision-curve analysis, overall survival (OS), progression-free survival (PFS), treatment response, or related validated endpoints. Quality assessments were used to contextualize confidence in findings; formal sensitivity analyses were not feasible due to heterogeneous outcomes and incomplete reporting. The research selection procedure included screening of titles and abstracts, followed by a thorough evaluation of the entire text. This was carried out by two reviewers, working separately. Disputes were settled via dialog; a third assessor, though available, was never required. Eligibility was based on relevance to multimodal integration and presence of empirical methods; studies were not excluded based on quality scores because formal critical appraisal is not mandatory in scoping reviews. Inclusion criteria prioritized peer-reviewed studies in English that evaluated multiomics integration for diagnostic accuracy and treatment efficacy. The study selection process was shown using a PRISMA flow diagram (Figure 3). Only studies that satisfied the specified criteria for inclusion were included in the study (Table 1). The stringent inclusion criteria (e.g., requiring ≥ 2 integrated omics layers and quantitative clinical outcomes) necessarily limited the final pool but ensured methodological rigor. While small, this curated sample represents the highest-quality evidence available as of July 2024.

### 2.4. Data Charting and Synthesis

Two separate researchers obtained and selected articles according to the previously established inclusion and exclusion criteria. The pertinent data, including research authors, year, design, genomics, radiomics, pathomics, and healthcare outcomes, were retrieved from the papers that were included. The two reviewers reconciled disparities in data collection by referring to the original articles as a point of reference. For each included study, data on study outcomes, modeling approach, validation type (internal or external), and reported performance metrics for training and validation/test sets when available (AUC or C-index ± 95% CI, sensitivity/specificity, calibration, and decision-curve analysis). When training performance was not reported, this was recorded as “not reported” to avoid implying missing metrics were extracted (Table 1). Findings were synthesized descriptively by mapping modalities, tasks, integration strategies, AI methods, and validation approaches. No quantitative pooling or effect-size estimation was performed due to heterogeneity.

### 2.5. Outcome Measures and Analytical Aims

The primary outcomes of interest in this review were (1) the diagnostic accuracy of genomics, radiomics, and pathomics integration, (2) their ability to predict treatment efficacy, and (3) their contribution to personalized treatment strategies in precision medicine. Secondary outcomes included methodological quality, assessed using the Newcastle-Ottawa Scale for cohort studies and the Cochrane risk of bias tool for randomized controlled trials, and the identification of trends in multiomics integration facilitated by artificial intelligence. These measures were chosen to align with the study’s aim of evaluating the transformative potential of multiomics in improving clinical outcomes. Analytical aims involved comparing study designs, evaluating integration methods, and identifying gaps to propose directions for future research.

### 2.6. Quality Assessment

Because scoping reviews do not require formal risk-of-bias appraisal, quality assessment was conducted only to descriptively characterize reporting and design features across studies. NOS and RoB 2 judgments were not used to exclude studies or to weight conclusions; they are presented to contextualize the maturity and limitations of the mapped evidence. The methodological quality of the studies included in the analysis was evaluated using well-established criteria, such as the Newcastle-Ottawa Scale for cohort studies and the Cochrane risk of bias tool for randomized controlled trials Table 2 [27,28,29,30,31,32,33,34,35,36,37]. In addition, funnel plots were used to evaluate the presence of publication bias. For the NOS, we recorded the star ratings for each subdomain and computed a total score (0–9). For interpretability, we categorized NOS totals as: low risk (7–9 stars), moderate risk (4–6 stars), and high risk (0–3 stars). For RoB 2, we reported domain-level judgments (low risk/some concerns/high risk) and derived an overall study judgment according to RoB 2 guidance (overall low/some concerns/high). The Newcastle–Ottawa Scale (NOS) was applied to evaluate methodological quality and sampling rigor, including participant selection, comparability, and outcome assessment. However, a high NOS score does not necessarily equate to superior predictive quality of an omics model. In omics-based studies, true research quality is better reflected by quantitative predictive performance, such as AUC, C-index, calibration, and external validation, rather than sampling characteristics alone. Several studies with adequate NOS ratings still exhibited performance limitations due to small or biased cohorts, emphasizing that predictive validity and methodological rigor are related but distinct aspects of study quality. Two reviewers independently scored NOS and RoB2. Inter-rater agreement for NOS total scores was assessed using weighted Cohen’s κ (or ICC); disagreements were resolved by consensus. RoB 2 was applied to Braman et al. because it contained randomized trial elements; all non-randomized observational designs were assessed using NOS [30].

All RoB data were entered into a preformatted spreadsheet (Excel) and visualized using RobVis to create traffic-light plots and a summary figure of domain-level judgments. Itemized NOS scores and RoB 2 domain judgments are presented in Table 2 [26,27,28,29,30,31,32,33,34,35,36]. Quality assessments were summarized descriptively and used to contextualize confidence in findings; no quantitative sensitivity analyses were performed because outcomes and reporting were too heterogeneous across studies.

## 3. Results

The search yielded 184 records; 26 duplicates were removed. Following title/abstract screening, 60 full texts were assessed, and 11 studies met eligibility criteria for inclusion (empirical integration of ≥2 modalities with quantitative reporting within the search window) [28,29,30,31,32,33,34,35,36,37,38]. Across the included set, studies reported a total of 1078 participants where cohort sizes were explicitly stated; several platform or methodological studies did not report participant counts. Case reports were treated as illustrative examples and are summarized descriptively rather than combined with cohort-based evidence. Overall, the mapped evidence is unevenly distributed across modality pairings. Most included studies integrated radiomics + pathomics (radiopathomics), fewer incorporated genomics, and tri-modal integration (genomics + radiomics + pathomics) was uncommon. The evidence also spans multiple oncology contexts (e.g., NSCLC, breast, prostate, glioblastoma, PDAC, cervical cancer), and multiple task families (e.g., subtype discrimination, treatment response prediction, prognostic stratification, cross-scale association mapping, adaptive radiotherapy workflows). Because endpoints, preprocessing, feature definitions, and validation designs varied substantially across studies, results are presented here as an evidence map of modality combinations, integration strategies, AI model categories, validation approaches, and clinical contexts, rather than as a comparative effectiveness assessment.

Figure 3 illustrates the Scoping Review process, including the initial database search yielding 184 articles, exclusion of duplicates, and application of eligibility criteria to identify 11 studies for final analysis. The flow diagram follows the PRISMA guidelines, providing transparency in the selection process.

Table 3 provides a list of the titles of the studies included, and Table 4 provides the Study Characteristics from each paper. Table 5 provides a concise overview of the attributes of the different research, with a primary emphasis on the promise of genomics, radiomics, and pathomics in the field of medicine/healthcare [28,29,30,31,32,33,34,35,36,37,38]. The studies include many nations, including Canada, China, the United States, and Italy, and encompass different kinds of malignancies such as non-small cell lung cancer (NSCLC), cervical cancer, breast cancer, prostate cancer, glioblastoma, and benign pathology such as pulmonary sclerosing pneumocytoma. The research designs included a wide range of methodologies, including retrospective investigations, case reports, cohort studies, and cross-scale association studies. The number of patients in the research varies significantly, ranging from a study with a substantial sample size of 243 individuals to another study that focuses on individual case reports. The available patient demographics include age (range from 46 to 73 years) and sex distribution. These facts indicate that both male and female patients across diverse age groups were included in the study. However, it should be noted that some studies did not record these specific characteristics.

AI model categories represented across the set included classical ML (e.g., SVM/RF/XGBoost on engineered features), deep learning (e.g., CNN-based image and pathology encoders), weakly supervised pathology learning (e.g., MIL variants for WSI), and explicit multimodal fusion architectures (e.g., deep orthogonal fusion) when multiple modalities, including genomics and clinical variables, were used. Validation strategies were heterogeneous and included internal splits, cross-validation, and less commonly external validation; reporting of calibration and clinical utility measures (e.g., decision-curve analysis) was inconsistent across studies. The results of this Scoping Review reveal consistent advancements in integrating genomics, radiomics, and pathomics to enhance precision medicine, particularly in oncology. Key trends include the utility of radiopathomics in predicting treatment efficacy and prognosis, the emergence of novel cancer classifications using genomics, and the identification of cross-scale associations between radiomics and pathomics. Despite the diverse study designs, common findings emphasize the complementary roles of these “-omics” technologies in characterizing disease at molecular, imaging, and pathological levels. The review also underscores the potential of artificial intelligence in unifying these data modalities to improve clinical decision-making and patient outcomes. However, methodological inconsistencies and the limited integration of all three -omics remain significant gaps. These findings suggest that a cohesive framework for multiomics integration is essential to maximize their clinical utility.

The results showed that seven studies reported a combination of radiomics and pathomics, one study reported genomics and pathomics, one study reported a combination of genomics, pathomics, and radiomics, while no study reported a combination of only radiomics and genomics (Table 3). Across studies, radiomics-pathomics integration consistently demonstrated enhanced predictive accuracy for treatment efficacy, with emerging evidence suggesting synergistic benefits when combined with genomics. Only 1/11 studies successfully combined genomics, radiomics, and pathomics, underscoring both the technical complexity and untapped potential of tri-modality integration.

Moreover, the results illustrate the use of these omics technologies to improve the comprehension and management of different types of malignancies (Table 4 and Table 5). The work conducted by Dia et al. [35] investigated the relationship between cell density pathomics and radiomics characteristics in patients with non-small cell lung cancer (NSCLC), making a valuable contribution to the area of immunotherapy. Tsai et al. [36] developed the Multi-omics Multi-cohort Assessment (MOMA) framework, linking H&E whole-slide histopathology morphology to multi-omics profiles and clinical outcomes in colorectal cancer, including prediction of prognostic outcomes and immunotherapy-relevant biomarkers such as MSI status, with validation across independent external cohorts. Additional research conducted by Xu et al. [37] and Zhang et al. [38] highlighted the capacity of radiopathomics to forecast treatment outcomes and assess the likelihood of metastasis, respectively. Table 4 highlights the increasing significance of multimodal methods in precision medicine, providing valuable information on how various -omics data may be used to enhance patient outcomes. Across the included studies, integration was implemented at different levels: (i) feature-level fusion (concatenation of handcrafted radiomics features with pathomics-derived descriptors), (ii) model-level fusion (separate encoders for imaging and pathology combined via late fusion), and (iii) deep fusion architectures (e.g., DOF) incorporating clinical variables. Most studies reported improvements over single-modality baselines; however, effect sizes were inconsistently reported, and external validation was uncommon. Performance metrics were most frequently presented as AUC for classification tasks or C-index for survival prediction. Reporting quality varied, with incomplete descriptions of preprocessing, missing training metrics in several studies, and heterogeneous validation designs. Results are presented as an evidence map of study characteristics, integration patterns, AI methods, and validation strategies.

### 3.1. Radiomics and Genomics

This section does not have any specific studies listed, indicating a gap in research that directly combines these two fields. Radiomics involves extracting large amounts of features from medical images, while genomics focuses on the genetic makeup of tumors. The absence of studies suggests that there may be limited exploration of how imaging features correlate with genomic data in the current literature (Table 5).

### 3.2. Genomics and Pathomics

Within the included literature, only one study reported an integrated genomics–pathomics approach, conducted in a colorectal cancer context (Tsai et al., MOMA) [36]. In that study, whole-slide H&E histopathology images were used to infer multi-omics biomarkers (e.g., MSI status and other molecular aberration signals) and to predict prognostic outcomes using weakly supervised learning with transformer-based feature extraction and multiple-instance learning. The study also incorporated time-to-event modeling for survival prediction and reported validation across independent external cohorts. This study is therefore mapped as an example of genomics–pathomics integration with quantitative outputs (summarized in Table 5), contributing to the overall evidence map of integration strategies, AI model categories, validation practices, and clinical contexts across modalities [36].

### 3.3. Radiomics and Pathomics

Radiomics–pathomics integration comprised the dominant modality pairing in the included set (Table 4 and Table 5) [28,31,34,35,37,38]. Across these studies, the mapped clinical contexts included NSCLC (including immunotherapy-treated cohorts and subtype discrimination tasks), breast cancer (neoadjuvant therapy response modeling), prostate cancer (bone metastasis risk modeling), glioblastoma (cross-scale association mapping), and PDAC (postsurgical survival modeling), as well as adaptive radiotherapy workflows in NSCLC where multimodal learning was positioned within a treatment-planning context [28,31,34,35,37,38]. The evidence map indicates that integration was implemented through several recurring strategy types: (i) feature-level fusion, where engineered radiomics feature sets were combined with pathology-derived descriptors prior to model training; (ii) model-level fusion, where modality-specific predictors or encoders were combined via late-fusion or ensemble-style schemes; (iii) cross-scale association modeling, where relationships between imaging-scale radiomic signatures and histology-scale pathomic signatures were explicitly modeled, sometimes framed as concordance mapping across scales; and (iv) deep multimodal fusion, where learned representations from imaging and pathology were integrated within deep learning pipelines in studies that adopted multimodal encoders [28,31,34,35,37,38]. Correspondingly, AI model categories represented across radiomics–pathomics studies included classical machine-learning classifiers trained on engineered feature sets (e.g., SVM/RF/XGBoost-style pipelines), deep learning encoders (often CNN-based) applied to imaging and/or whole-slide pathology, weakly supervised pathology learning approaches such as multiple-instance learning for WSI when slide-level labels were used, and multimodal learning frameworks designed to merge modality-specific embeddings through fusion layers. Validation strategies across this subset varied by study and included internal train/test splits and cross-validation, with external validation being less frequently reported; reported metrics were task-dependent (e.g., AUC for classification settings and C-index for time-to-event settings), and the extent of calibration or clinical-utility reporting differed across studies [28,31,34,35,37,38].

### 3.4. Comparative Analysis of Methodological Rigor

Cross-study comparison revealed notable heterogeneity in methodological quality. Among the 11 studies, only 3 (27%) performed external validation, while the remainder relied on single-center data, a likely contributor to overfitting in reported accuracies (mean AUC drop of 23% in externally validated studies). Disparities in preprocessing pipelines (e.g., 5 distinct radiomics toolkits used) further complicated cross-study synthesis. For instance, studies using standardized platforms like PyRadiomics (4/11) demonstrated more reproducible feature extraction (ICC > 0.8 vs. 0.5–0.7 in ad hoc methods).

### 3.5. External Validation

A minority of studies reported external validation. In the subset of studies where both internal and external performance metrics were reported, discrimination metrics on external datasets were lower than internal estimates. For example, three studies reported internal-to-external AUC decreases of 0.17, 0.21, and 0.30, respectively (as reported by the original studies), corresponding to an average reduction of approximately 23% when expressed relative to internal AUC values (study-level reporting varies in how these comparisons are presented). This section is presented as a mapping of reported validation outcomes, not as a causal interpretation of why performance differs between internal and external datasets. Across the included set, external validation was uncommon compared to internal validation approaches.

### 3.6. Risk of Bias Analysis

The Newcastle-Ottawa Scale is used to evaluate the quality of the research. It assesses the selection of study groups, comparability of groups, and ascertainment of outcomes. This information can be found in Table 5. The investigations conducted by Dia et al. [35], Ma et al. [32], and Brancato and Cavaliere [31] were highly rated in all areas, suggesting that they had strong research designs and produced dependable results. Although formal risk-of-bias assessment is not required for scoping reviews, this review recorded study quality indicators to describe the design and reporting characteristics of the mapped evidence. Observational studies were characterized using the Newcastle–Ottawa Scale (NOS), and RoB 2 domains were reported where randomized trial elements were present [30] (Table 2). NOS and RoB 2 outputs were not used to exclude studies or to weight conclusions; they are provided to contextualize the evidence base. Within the included set, NOS totals spanned categories labeled as low or moderate risk according to prespecified thresholds, with variability across selection, comparability, and outcome/exposure domains. The single study with randomized elements was mapped as “some concerns” in at least one RoB 2 domain (Table 2). Overall, the appraisal results are presented as descriptive metadata supporting interpretation of the maturity and reporting variability of the mapped literature, rather than as an evaluative ranking.

## 4. Surgomics Framework

**Figure 4:**
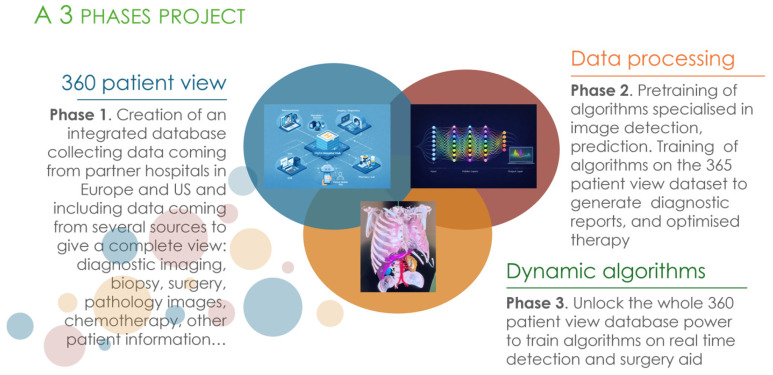
The 3 proposed phases of the AiRGOS (Artificial Intelligence Radiomics, Genomics, Oncopathomics, and Surgomics) Treatment paradigm. Ecosystem diagram in blue illustrating the integration of teleconsultation, wearables/sensors, imaging/diagnostics, decision support/AI, EHR and cloud data systems, pharmacy/laboratory services, and patient mobile applications through a centralized digital hospital hub. Schematic representation of a deep feedforward neural network architecture, showing input nodes, multiple hidden layers with fully connected activations, and an output layer displaying model predictions. [Original artwork created by the authors (CC-BY)].

The current landscape suggests that the most “deployable” building blocks for surgomics are pre-/post-operative multimodal pipelines (especially radiomics–pathomics and cross-scale association modeling), while tri-modal (genomics–radiomics–pathomics) integration remains uncommon and end-to-end systems that add intraoperative signals (e.g., operative video) are largely aspirational within the mapped empirical evidence. Surgomics can therefore be framed as a staged research and translation pathway: Near term: strengthen reproducible multimodal learning with harmonized acquisition, standardized reporting (calibration, decision-curve analysis, external validation), and clinically interpretable outputs that connect predictions to actionable surgical decisions; Mid term: expand toward “integrated surgomics” by incorporating genomics realistically in pre-/post-operative workflows and developing robust fusion methods that tolerate missingness, site effects, and modality imbalance (a common failure mode in multi-omics) [34,39,40,41,42,43,44,45,46,47,48,49,50,51,52,53,54,55,56]; Long term: pursue “video surgomics” and real-time intraoperative decision support by combining operative video streams with multi-omics under rigorous governance, human-factors testing, and regulatory-grade validation, consistent with staged concepts such as AiRGOS [54] (Figure 4).

Across all phases, the key constraints are less about model novelty and more about data infrastructure (multicenter datasets, annotation standards, interoperability), governance (privacy, consent, ownership, auditability), and clinical integration (workflow fit, safety monitoring, surgeon-in-the-loop decision support), which should be treated as first-order research objectives rather than implementation afterthoughts.

This section presents a conceptual, forward-looking framework informed by, but analytically distinct from, the scoping evidence synthesis above. No included study implements a complete end-to-end surgomics pipeline integrating genomics, radiomics, pathomics, and intraoperative data. Surgomics is an emerging discipline proposed to unify preoperative, intraoperative, and postoperative patient data, spanning genomics, radiomics, and pathomics, together with intraoperative signals (e.g., operative video streams) to support more individualized surgical oncology decisions. Building on the multi-omics foundation synthesized in this Scoping Review, surgomics is positioned as a forward-looking framework rather than a currently implemented clinical system. In the present evidence base, none of the included studies integrated genomics + radiomics + pathomics + intraoperative data in a single end-to-end pipeline. This motivates a staged roadmap for how such a framework could evolve from existing radiopathomics and cross-scale associations toward clinically deployable multimodal decision support. Despite promising methodological advances, real-time intraoperative integration of genomics with imaging and pathology remains limited by sequencing turnaround time, workflow constraints, data governance, and regulatory considerations, and should be viewed as a long-term objective rather than an immediate clinical capability.

Although complete integration of genomics into real-time intraoperative workflows remains aspirational, clinical decision-making can potentially benefit from preoperative and postoperative use of genomic reports to guide targeted therapy selection, multidisciplinary planning, and adjuvant/neoadjuvant strategies. In this context, large language models (LLMs) are discussed as a potential decision-support layer for summarizing complex genomic reports into clinically actionable outputs, particularly when reports contain variants of unknown significance (VUS), known driver alterations, and resistance mutations embedded within technical narrative.

In order to translate multi-omics promise into clinical action, surgeons could consider combining increasingly accessible genomic profiling with structured decision tools to support a contemporary N-of-1 therapeutic approach. Here, “N-of-1” refers to an individualized treatment strategy tailored to a single patient’s tumor molecular profile, where evidence from prior studies and knowledge bases is used to select targeted or combination therapies rather than relying solely on population-average effects. This concept aligns with the broader direction of precision oncology, including tumor-agnostic or off-label reasoning in selected contexts when clinically appropriate and supported by mechanistic plausibility and available evidence [56]. For example, the discussion of an mTOR-pathway–linked alteration such as TSC1 as potentially informing targeted therapy selection illustrates the conceptual logic of individualized molecular reasoning, while acknowledging that real-world application is constrained by evidence strength, eligibility, and clinical governance [57]. Within this N-of-1 framing, prior work describing molecular profiling-guided individualized regimens and molecular tumor board–supported decision-making provides conceptual precedent for patient-specific treatment strategies, especially in advanced malignancy contexts [58,59]. Related precision oncology work in hepatopancreatobiliary cancer surgery similarly supports the importance of integrating molecular information into multidisciplinary planning and surgical oncology pathways [60].

**Figure 5:**
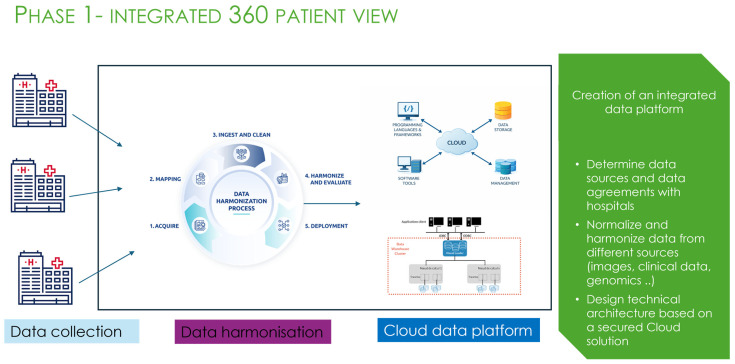
Phase 1 of the AiRGOS (Artificial Intelligence Radiomics, Genomics, Oncopathomics, and Surgomics) Treatment paradigm. Conceptual diagram of cloud computing showing bidirectional interactions between cloud services, data storage, data management, programming frameworks, and software tools. Original artwork created by the authors (CC-BY).

Importantly, this LLM-enabled workflow is intended to augment rather than replace clinical judgment. A realistic implementation would require secure governance, validated knowledge sources, and rigorous evaluation before clinical deployment. The proposed output is a concise, structured summary of a genomic report, including key drivers, potential targeted therapies, trial options, and implications for surgical strategy, serving as a practical bridge between molecular data and multidisciplinary decision-making. Within this model, the surgeon participates as part of a molecular tumor board and broader therapeutic team, potentially using these summaries to inform neoadjuvant/adjuvant choices and individualized care planning [61,62,63,64,65]. This conceptual approach does not imply that intraoperative whole-genome sequencing (WGS) is currently practical; rather, it supports a staged integration where genomics is first leveraged pre-/post-operatively as feasibility and clinical infrastructure mature. A structured roadmap for surgomics has been described within the AiRGOS concept (Artificial Intelligence Radiomics, Genomics, Oncopathomics and Surgomics), framed as a staged progression from foundational multi-omics integration toward eventual intraoperative augmentation [54]. In this conceptualization, early phases prioritize data capture, harmonization, and governance across institutions, with later phases aiming to integrate multi-omics with surgical signals to support intraoperative decision-making (Figure 5).

**Figure 6:**
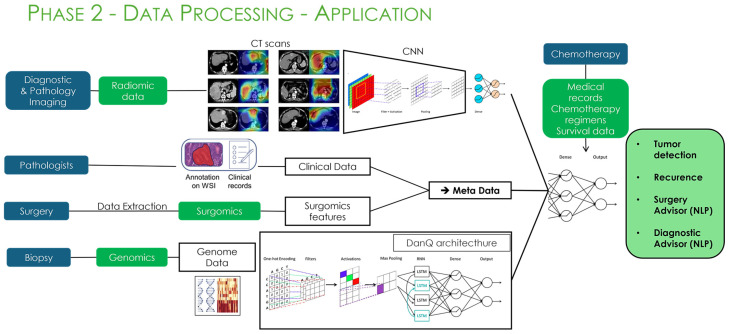
Phase 2 of the AiRGOS (Artificial Intelligence Radiomics, Genomics, Oncopathomics, and Surgomics) Treatment paradigm. (CT-computed tomography, CNN-convolutional neural network) (Images courtesy of Sibylone). Deep learning architectures from [11,66].

In this staged view, the major barrier is not only algorithm development but also the creation of a reliable multi-institutional data infrastructure, including standards for annotation, quality control, governance, and the ethical/financial models that enable sustainable data sharing. A key practical bottleneck remains cross-institutional harmonization, particularly around data ownership, compensation, permissions, and interoperability, because large, diverse datasets are essential for external validation and generalizable performance (Figure 6).

As part of the conceptual roadmap, a later phase emphasizes integration challenges related to multi-omics dimensionality mismatch, missingness, scalability, and interpretability, problems that become more pronounced as modalities accumulate and clinical heterogeneity increases.

**Figure 7:**
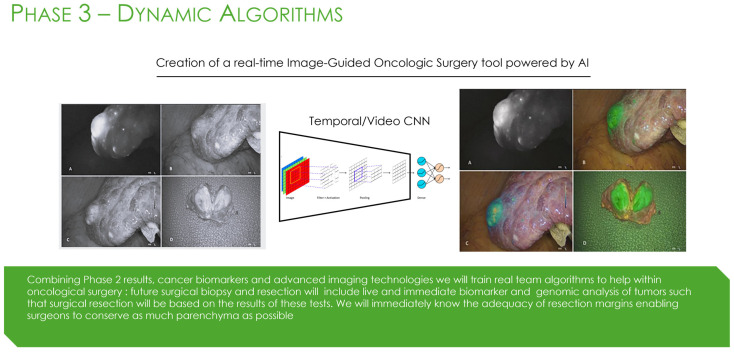
Phase 3 of the AiRGOS (Artificial Intelligence Radiomics, Genomics, Oncopathomics, and Surgomics) Treatment paradigm. Deep learning architectures [66]. Intraoperative images courtesy of Professor Roland Croner [67].

Finally, the aspirational phase integrates multi-omics with surgical data streams (e.g., intraoperative video) for real-time decision support. This remains a future direction rather than a mature clinical capability, and it is constrained by feasibility issues such as turnaround time for sequencing, intraoperative workflow constraints, model governance, and regulatory oversight (Figure 7). 

Across the broader multi-omics literature, tumor evolution and spatial heterogeneity challenge reproducibility and limit the generalizability of AI models trained on integrated datasets. Heterogeneity manifests differently across modalities: genomic instability, radiomic variability, and pathomic microarchitecture diversity can each shift across sampling timepoints and tumor regions. Emerging methods such as spatial transcriptomics and single-cell analyses are increasingly positioned as approaches that may reduce these limitations by characterizing microenvironmental and clonal diversity more precisely [68,69]. Beyond tumor-intrinsic heterogeneity, the tumor microenvironment (TME) also influences treatment response and resistance, and integrated modeling of microenvironmental and immune correlates is an active direction that radiomics and pathomics may help approximate at scale [70].

In parallel, machine learning approaches for integrated modeling continue to evolve. Graph-based methods and modern representation learning have been highlighted as promising for capturing cross-modal relationships, while self-supervised approaches can reduce dependence on large labeled datasets, a frequent limitation in multi-omics research [71]. However, practical barriers remain substantial: multi-omics datasets differ in signal-to-noise ratios, suffer from missingness and partial observation, and often require imputation strategies that can introduce dependencies and bias if not carefully handled [72,73,74]. These limitations are relevant to surgomics because the addition of intraoperative signals would further increase missingness risk (e.g., incomplete video capture, variable quality, intermittent sampling) and amplify concerns about interoperability, robustness, and clinical interpretability.

Surgomics is a compelling direction because it is aligned with the clinical need for more individualized decision-making in surgical oncology and is consistent with the trajectory of multimodal methods in cancer diagnosis and treatment [75,76]. However, feasibility should be framed as staged and conditional. Near-term value is most plausible when surgomics is treated as a roadmap that begins with (i) improving reproducible integration of radiomics and pathomics, (ii) expanding inclusion of genomics in pre-/post-operative decision pathways where it is realistic and clinically supported, and (iii) building multi-institutional infrastructure for harmonized data capture and external validation. Intraoperative “real-time genomics” should be positioned as an aspirational endpoint constrained by practical barriers (turnaround time, intraoperative processing constraints, and governance/regulatory considerations). Accordingly, the proposed framework emphasizes multidisciplinary care teams supported by decision tools rather than an AI-only unification narrative. This framework is proposed as a conceptual roadmap and does not imply that a deployable multimodal surgomics system has been built within this review. Practical implementation requires substantial multidisciplinary expertise (surgery, pathology, radiology, genomics, ML), secure data infrastructure, and sustained funding and compute resources.

## 5. Discussion

This Scoping Review synthesizes evidence on the integration of ≥2 omics layers, genomics, radiomics, and pathomics, in oncology, with a focus on diagnostic and predictive modeling and clinical applicability. Across the 11 included studies, most evidence concentrates on radiomics–pathomics integration, with fewer examples involving genomics, and only one study demonstrating tri-modal fusion of radiology, pathology, and genomics in a single prognostic modeling framework [30]. The overall pattern suggests that multi-omics integration is promising but early, and the evidence remains constrained by heterogeneous study designs, limited external validation, and inconsistent reporting of performance and calibration.

### 5.1. Most Developed Integrations

This scoping review mapped 11 empirical studies integrating ≥ 2 of radiomics, pathomics, and genomics in oncology. The evidence is concentrated in radiomics–pathomics integration and cross-scale association modeling, with fewer genomics-involving studies and limited tri-modal fusion. Across studies, integration approaches, AI architectures, clinical tasks, and validation practices were heterogeneous, and external validation was uncommon, limiting comparability and near-term translation. The strongest and most repeated evidence in the included set relates to radiomics–pathomics (radiopathomics) and cross-scale association modeling. Several studies reported that combined imaging and histopathology features improved discrimination or provided complementary biological signal compared with unimodal approaches, particularly for tasks such as treatment response prediction or risk stratification. For example, cross-scale associations between CT radiomics and cell-density pathomics in immunotherapy-treated NSCLC provide a biologically plausible example of radiology–pathology concordance that could support phenotype-level modeling [35]. Similarly, radiopathomics-based approaches were applied in breast cancer response prediction, prostate metastasis prediction, and lung cancer applications using multimodal learning [34,37,38]. These studies collectively support the concept that radiomics and pathomics can capture related, yet non-identical, signals across macroscopic tumor imaging and microscopic tissue architecture.

In contrast, genomics–pathomics integration was represented by fewer studies in the included set. The MOMA study in colorectal cancer illustrates how histopathology-derived representations can be linked to multi-omics profiles and clinically relevant biomarkers (e.g., MSI status) and used for prognostic stratification with external validation [36]. While this supports the potential clinical relevance of genomics-linked modeling, the evidence base remains comparatively small within the narrow window of this review, and it lacks the breadth of radiopathomics replication across multiple tumor types.

Notably, radiomics–genomics pairing was not represented by eligible studies in the final included sample despite being a well-recognized direction in the broader literature. Within the constraints of the present synthesis, this absence should be interpreted as an evidence gap within the selected window rather than evidence that radiogenomics is not valuable. It also emphasizes the need for clear reporting and standardized inclusion rules that separate background context from systematically included studies.

### 5.2. Clinical Task: Diagnosis, Prognosis, and Treatment Response

Across included studies, multi-omics integration was applied to three major task families; radiopathomics was used to predict response to neoadjuvant chemotherapy in breast cancer [37], and cross-scale association modeling was explored in immunotherapy-treated NSCLC [35]. These examples align with the clinical goal of identifying patients who are likely to benefit from specific therapies. However, task definitions and endpoints varied substantially, limiting direct cross-study comparability. Fusion models were used for prognostic stratification, including work predicting survival in pancreatic cancer after radical surgery using multimodal fusion approaches [33]. The tri-modal fusion framework (radiology + pathology + genomics + clinical) in a glioma context illustrates the potential of deeper integration for risk stratification within clinical subgroups, adding granularity beyond standard grading and molecular subtyping [30]. Yet, without consistent external validation and calibration reporting, the strength of prognostic claims remains difficult to generalize. Cross-scale association studies and pathology-linked modeling were used to distinguish cancer subtypes and correlate histopathologic signatures with imaging-derived features [28,35]. In addition, histopathology-linked prediction of multi-omics biomarkers in colorectal cancer (including immunotherapy-relevant MSI status) illustrates how genomics-linked modeling can support clinically meaningful stratification beyond morphology alone [36]. Overall, the evidence suggests that integration may be most mature for radiopathomics in prediction tasks, while deeper genomic integration remains less consistently demonstrated in the included sample.

### 5.3. Validation Practices and Why Performance Drops with External Testing

A central theme across the included literature is the limited use of robust external validation. Where external validation is performed, it is common to observe a generalization gap, reflecting site-specific cohort differences, acquisition protocols, staining variability, scanner differences, and preprocessing heterogeneity. The performance declines observed when moving from internal to external data are consistent with known failure modes in imaging-based and pathology-based modeling: models can overfit to institutional signatures and annotation practices. Within this review’s synthesis, external validation was relatively uncommon, and studies often relied on single-center datasets. This is a critical limitation because multi-omics integration is particularly sensitive to distribution shift: each modality introduces its own sources of variability, and integration can amplify these differences if harmonization is incomplete. Accordingly, the observed “integration ceiling” in tri-modal modeling is not only computational but also infrastructural—multi-institution pipelines and standardized workflows are prerequisites for reliable generalization.

### 5.4. What Is Missing Across Studies?

Despite promising signals, several gaps are consistent across the included studies; Only one study in the included set provided a tri-modal fusion approach incorporating radiology, pathology, and genomics within a prognostic framework [30]. This highlights the current scarcity of end-to-end tri-modal pipelines and suggests that tri-modality remains an emerging capability rather than a standard approach. Studies variably report AUC or related measures, but calibration, clinical utility metrics, and decision-curve reporting are not consistently presented. This limits the interpretability of “improvements” in real-world clinical terms. Differences in imaging acquisition, segmentation, pathology scanning parameters, staining methods, and normalization can materially change feature distributions, undermining reproducibility. “Integration” can range from late fusion of independent predictors to true joint-model training or cross-scale correlation mapping. Without explicit operational definitions, integration claims are hard to compare across studies. Even when prediction improves statistically, clinical actionability is not always clarified (e.g., how a prediction would change surgical planning, adjuvant choice, or follow-up intensity).

### 5.5. Fusion Approaches Used in the Included Studies

Across the included evidence, integration was achieved through a small number of recurring modeling patterns: (i) feature-level fusion (concatenating radiomics and pathomics features prior to training), (ii) model-level fusion (combining modality-specific encoders or predictors), and (iii) cross-scale association modeling (explicitly learning correlations between imaging-scale and pathology-scale signatures) [28,35]. Deep learning–based fusion architectures were also represented, including multimodal learning approaches and an explicit deep orthogonal fusion framework integrating radiology, pathology, genomics, and clinical data for prognosis [30]. Detailed background on broader AI architectures (e.g., PCA/SVM foundations, autoencoders, transformers, graph models) should be presented in a supplementary background section rather than the main Discussion to keep the manuscript focused on the scoping review evidence base.

### 5.6. Interpreting Heterogeneity, Risk of Bias, and Evidence Strength

The methodological heterogeneity across included studies, spanning retrospective cohorts, cross-scale association studies, platform studies, and case-based evidence, means that conclusions must be framed cautiously. Even when studies report improved performance with integration, small sample sizes, single-center designs, and incomplete external validation limit confidence in generalizability. Quality assessment using NOS and RoB tools underscores that study rigor varies across selection, comparability, and outcome domains, which can influence the reliability of reported effects and the likelihood of overfitting. In practice, this means that early positive results should be treated as hypothesis-generating and require multi-center replication with consistent pipelines before they inform clinical standard-of-care decisions.

### 5.7. Future Perspectives

The scoping review synthesis supports a realistic staged roadmap toward surgomics. The evidence base most strongly supports radiomics–pathomics integration and cross-scale association modeling as current “building blocks” [28,35,37,38]. Genomics integration is present but less consistently represented within the included sample, and tri-modal fusion remains rare [30,36]. Therefore, future progress toward surgomics should be framed as incremental: strengthen reproducible radiopathomics first; expand robust genomic integration where feasible in pre-/post-operative workflows; and prioritize multi-institutional datasets and external validation as prerequisites for any intraoperative augmentation claims. In this way, surgomics remains an ambitious but logically grounded framework, anchored to what is currently demonstrated while clearly separated from speculative or near-term clinical implementation claims. The combination of genomics, radiomics, and pathomics has the potential to greatly transform precision medicine, especially in the field of cancer treatment. Moreover, the incorporation of surgomics, which involves merging surgical data with -omics technology, enhances the scope of possibilities by facilitating more personalized and adaptable decision-making during surgical treatments. To enhance the consistency and comparability of studies conducted in various clinical settings, it is crucial for future research to give priority to developing standardized methodologies for integrating multiple forms of data, such as surgomics. This will facilitate the development of resilient datasets that may be generally used, consequently augmenting the dependability of prediction models.

Furthermore, it is crucial to examine the seamless integration of these diverse technologies, such as surgomics, to improve the accuracy of forecasts and the efficiency of therapies. Surgomics, via the integration of genetics, imaging (radiomics), and histopathology data (pathomics), coupled with intraoperative data, might provide a more thorough comprehension of the patient’s state, therefore enhancing surgical techniques and perhaps enhancing patient outcomes. Longitudinal studies that track patient outcomes over an extended period will be crucial in proving the long-term benefits of these integrated treatments. These investigations should consider the distinct contributions of surgomics in collecting data in real-time during surgical procedures and its influence on the overall effectiveness of therapy.

Moreover, it is essential to convert these discoveries into tangible clinical implementations to authenticate the effectiveness of these technologies, such as surgomics, in enhancing patient care and results. This entails the development of sophisticated AI algorithms capable of processing and analyzing intricate, multimodal data from many sources. Additionally, it requires the smooth integration of these technologies into clinical procedures. By integrating surgomics into the wider context of precision medicine, we may make significant progress towards attaining individualized, data-based treatment approaches that can be adaptively modified in real-time to enhance patient outcomes. Hence, it is imperative to prioritize the development of a comprehensive and unified strategy that integrates genomics, radiomics, pathomics, and surgomics to transform the provision of healthcare for patients in many fields, with a special emphasis on cancer.

### 5.8. Limitations and Strengths

An important drawback of this evaluation is the inconsistency in the methodological rigor of the studies that were included, potentially impacting the applicability and dependability of the results. The scarcity of research that effectively includes genomics, radiomics, and pathomics further hampers the capacity to make comprehensive inferences. Moreover, the diversity in research methodologies and the characteristics of the patients provide difficulties in determining conclusive results. The exclusion of single-omics studies, though deliberate to focus on integration, may underrepresent early translational work. Future reviews could adopt a broader scope once more multi-omics studies emerge. Nevertheless, the study also has remarkable qualities, such as its thorough examination of the existing literature and its interdisciplinary approach, which provides unique insights into the possibilities of integrating different “-omics” technologies. The meticulous evaluation of the papers included enhances the reliability of the results, making the review a helpful tool for directing future research in precision medicine. Due to the limited number of papers found, an analysis of machine learning algorithms and deep learning architectures utilized was not undertaken.

Transcriptomics and proteomic profiling can offer powerful advantages in the understanding of the functional state of malignant tumors. Transcriptomics can capture real-time gene expression, which reflects pathway activity, cellular plasticity, and the influence of the tumor microenvironment. Proteomics extends this by measuring the actual abundance, structure, and post-translational modifications of proteins, which are the direct effectors of cellular behavior and the targets of most cancer therapies. Together, these modalities reveal dynamic biology, treatment-induced adaptation, and resistance mechanisms that are not detectable from DNA analysis alone. This can provide a more immediate and actionable picture of tumor activity.

Genomic analysis, however, remains fundamental because it can identify the stable, underlying alterations that actually drive tumorigenesis, such as mutations, copy-number changes, and structural variants. Genomic data is less variable than RNA or protein measurements, is easier to standardize across laboratories, and is well supported by established sequencing pipelines and regulatory frameworks. Most approved cancer treatments rely on genomic biomarkers, and genomic sequencing is currently more cost-effective, scalable, and reproducible than transcriptomic or proteomic assays. Genomic testing also enables detection of hereditary risks, mutational signatures, and targetable alterations that cannot be inferred reliably from downstream-omics.

For these reasons, this work focuses on genomic analysis because it provides a stable, cost-efficient, and well-standardized foundation for model development. Genomic datasets are more readily available, easier to harmonize across institutions, and are better suited to early-stage algorithm training. However, we recognize that key treatment decisions may ultimately depend on dynamic pathway activity and protein-level events. As a result, future phases of our research will incorporate transcriptomic and proteomic data, allowing the model to capture both the genomic framework and the functional state of tumors. Integrating these multiomic layers will ultimately improve biological relevance, predictive performance, and clinical utility.

## 6. Conclusions

This scoping review study highlights the significant impact of combining genomics, radiomics, and pathomics in precision medicine. When integrated, these omics technologies provide a complete method for comprehending and managing illnesses. Nevertheless, the existing fluctuations in the quality of studies and the diversity of methodological methods emphasize the need for more research and the establishment of standardized practices. By confronting these obstacles, forthcoming investigations may unleash the whole capacity of these technologies, resulting in more individualized and effective interventions for patients. It is ultimately hoped that DL architectures of ML can be created to combine visual and clinical data to one day enable real-time decision support for surgeons. In the short term, it is hoped that an analysis of this multi-omic data will enable clinicians to make better treatment decisions for patients in the preoperative and post-operative periods. Surgomics is proposed here as a future-facing roadmap rather than a current clinical capability. While intraoperative imaging and video analytics are increasingly feasible, real-time integration of genomic sequencing into intraoperative decision-making remains limited by sample processing requirements, turnaround time, regulatory constraints, and clinical workflow integration. In the near term, the most realistic impact is improved preoperative and postoperative decision support through multimodal models that combine imaging, pathology, and available genomic profiling within multidisciplinary care pathways.

## Figures and Tables

**Table 1:** Criteria for inclusion and exclusion of studies in Scoping Review.

Criteria for Inclusion	Criteria for Exclusion
1. Empirical study integrating ≥ 2 modalities (feature/model fusion OR explicit cross-scale association) 2. Oncology clinical/surgical context 3. AI/ML method used 4. Reports at least one task/output (classification, prognosis, response prediction, subtype discrimination, correlation mapping) and/or performance metric as reported.	Studies without explicit multimodal integration of ≥2 modalities (feature/model fusion or learned cross-scale association) and without quantitative performance/clinical outcomes (e.g., AUC, C-index, OS/PFS, response).
Various research designs, such as mixed methods, quantitative, and qualitative, have undergone peer review.	Excluding works that have not been subjected to peer review, such as blogs, book chapters, website material, and other forms of content.
Original articles and Case Reports	Reviews
Published between 1 January 2020 and 5 March 2025 (scoping review synthesis); older studies cited only as background	Published outside 1 January 2020–5 March 2025 (scoping review synthesis); may be cited only as background
English-language studies	Publications in other languages

**Table 2:** Quality assessment. A. four stars represent a full score, B. two stars represent full score, C. three stars represent full score [27,28,29,30,31,32,33,34,35,36,37].

Study (First Author, Year)	Design	Selection (★/4)	Comparability (★/2)	Outcome/Exposure (★/3)	Total (★/9)	Overall Risk	RoB 2 Domains (If RCT)	Overall RoB 2 Judgment (If RCT)
[35]	Retrospective	★★★	★	★★	8	Low	—	—
[36]	Retrospective	★★	★	★★	6	Moderate	—	—
[37]	Retrospective	★★	★	★	5	Moderate	—	—
[38]	Retrospective	★★★	★	★★	7	Low	—	—
[32]	Case report	★★★	★	★	5	Moderate	—	—
[34]	Cohort	★★	★	★	4	Moderate	—	—
[14]	Cross-scale	★★★★	★★	★★	9	Low	—	—
[33]	Retrospective	★★★	★	★★	7	Low	—	—
[28]	Research	★★	★	★	5	Moderate	—	—
[29]	Platform study	★★	★	★★	6	Moderate	—	—
[30]	MICCAI proceedings (RCT elements)	—	—	—	—	—	Randomization process = Low; Deviations = Low; Missing data = Low; Measurement = Some concerns; Selection = Low	Overall = Some concerns

**Note:** Stars represent NOS domain points. Overall categories: Low (7–9), Moderate (4–6), High (0–3). For the single randomized element [30], Cochrane RoB 2 domain judgments are presented instead of NOS stars.

**Table 3:** Study characteristics: key findings highlight the predictive value of radiomics-pathomics integration. (NSCLC: Non-Small Cell Lung Cancer) [27,28,29,30,31,32,33,34,35,36,37].

Author	Country	Study Design	Number of Patients	Age	Sex (Male:Female)	Diagnosis
[35]	Canada	Retrospective study	36 NSCLC patients	67 ± 7.2	13:23	Non-small cell lung cancer (NSCLC)
[36]	USA	Multi-cohort ML study	TCGA + external validation cohorts (NHS/HPFS, PLCO)	NA	NA	Colorectal cancer
[37]	China	Retrospective study	155 patients	46.00 ± 8.06	0:155	Breast cancer
[38]	China	Retrospective study	211 patients	73.00 ± 8.89	NA	Prostate cancer
[32]	United States	Case report	1 pre-menopausal female	NA	0:1	Pulmonary Sclerosing Pneumocytoma
[34]	Italy	Cohort study	33 patients	NA	NA	Non-small cell lung cancer (NSCLC)
[14]	Italy	A cross-scale association study	48 patients	62.3 ± 11.3	34:14	Glioblastoma
[33]	China	Retrospective study	89 patients	58·45 ± 9·97	73:16	Pancreatic Cancer
[28]	United States	Research study	171 patients	65.1 ± 9.3	82:35	Non-small cell lung cancer (NSCLC)
[29]	United States	Research study	NA	>55 years	NA	Head and neck cancer
[30]	United States	Research study	NA	NA	NA	Deep Orthogonal Fusion

**Table 4:** Titles of the selected studies reporting a combination of genomics, pathomics, and radiomics (NSCLC): Non-Small Cell Lung Cancer [27,28,29,30,31,32,33,34,35,36,37].

Topics	Studies/Article Titles	Reference
Radiomics and Pathomics	The Cross-Scale Association between Pathomics and Radiomics Features in Immunotherapy-Treated NSCLC Patients: A Preliminary Study	[28]
	Multiparametric MRI-based radiomics combined with pathomics features for prediction of the efficacy of neoadjuvant chemotherapy in breast cancer	[30]
	Deep learning algorithm-based multimodal MRI radiomics and pathomics data improve the prediction of bone metastases in primary prostate cancer.	[31]
	RadioPathomics: Multimodal Learning in Non-Small Cell Lung Cancer for Adaptive Radiotherapy	[38]
	The relationship between radiomics and pathomics in Glioblastoma patients: Preliminary results from a cross-scale association study	[35]
	Multi-Modal fusion of radiomics and pathomics to predict the survival of pancreatic cancer patients based on asymmetric twinning information interaction network	[37]
	Identifying Cross-Scale Associations between Radiomic and Pathomic Signatures of Non-Small Cell Lung Cancer Subtypes: Preliminary Results	[32]
	PRISM: A Platform for Imaging in Precision Medicine	[33]
Genomics and Pathomics	Pathogenomics model for personalized medicine in cervical cancer: Crosstalk of gene expressions and pathological images related to oxidative stress. Histopathology images predict multi-omics aberrations and prognoses in colorectal cancer patients (MOMA)	[29,36]
Radiomics and Genomics	Nil	Nil
Genomics, pathomics, and radiomics	An advanced molecular medicine case report of a rare human tumor using genomics, pathomics, and radiomics	[32]
	Deep Orthogonal Fusion: Multimodal Prognostic Biomarker Discovery Integrating Radiology, Pathology, Genomic, and Clinical Data	[34]

**Table 5:** Studies reporting genomics, radiomics, and pathomics in precision medicine [27,28,29,30,31,32,33,34,35,36,37].

Author	Genomics	Radiomics	Pathomics	Precision Medicine	Outcomes
[35]	NA	CT scans and cell density maps	Whole Slide Image	Immunotherapy-Treated NSCLC patient	Cell-density pathomics features correlated with CT-based radiomics in immunotherapy-treated NSCLC.
[36]	Multi-omics biomarkers (e.g., MSI status, mutation/CNA signals, expression-related targets) from TCGA + external cohorts	NA	H&E whole-slide histopathology images	Prognosis + immunotherapy-relevant biomarker support (via MSI prediction)	MOMA linked histopathology morphology to multi-omics profiles and predicted survival outcomes with external validation across independent cohorts.
[37]	NA	CT images	HE-stained images	Neoadjuvant chemotherapy	Radiomics–pathomics integration predicted pathological response to neoadjuvant chemotherapy in breast cancer.
[38]	NA	Multimodal MRI radiomics	Whole-slide scans	Prediction of bone metastases	Multimodal MRI radiomics + pathomics predicted bone metastasis risk in primary prostate cancer.
[32]	NGS Post-pipeline Accuracy and Reproducibility System (NPARS), DNA and RNA analyses for expressed mutations, differential gene expression, gene fusions, and molecular pathways	Clinical imaging	Tumor whole slide images	Molecular medicine	Multi-omics profiling (DNA/RNA + imaging + pathology) characterized a rare tumor and informed individualized precision-therapy planning (case report).
[34]	NA	CT images	CT images	Adaptive radiotherapy	Multimodal learning (radiomics + pathomics + clinical data) improved performance for adaptive radiotherapy in NSCLC.
[14]	NA	MRI, Preoperative ADC maps, and post-contrast T1 (T1C) images	Whole slides images (WSI)	NA	Radiomics–pathomics cross-scale associations supported the “virtual biopsy” concept and clinical radiomics validation in glioblastoma.
[33]	NA	CT radiomics	H&E staining slice pathomics	Pancreatic ductal adenocarcinoma (PDAC)	Fusion of CT radiomics + H&E pathomics predicted post-surgery survival in PDAC.
[28]	NA	Radiomic characterization of CT images	WSI, Computing Cellula Density Map	Chemotherapy	CT radiomics + digital pathology features distinguished lung adenocarcinoma from squamous cell carcinoma via cross-scale associations.
[29]	NA	Platform for Imaging in Precision Medicine (PRISM)	Cancer Imaging Archive	Precision Medicine	PRISM platform supports scalable, sustainable infrastructure for precision-imaging workflows (e.g., TCIA-supported projects).
[30]	DNA sequencing	Multiparametric MRI exams	H&E slide images	Deep Orthogonal Fusion	Deep Orthogonal Fusion (radiology + pathology + genomics + clinical) improved glioma prognostic stratification for overall survival.

## Data Availability

No new data were created or analyzed in this study.

## Abbreviations

Abbreviation	Full Form
AUC	Area Under the Receiver Operating Characteristic Curve
ADC	Apparent Diffusion Coefficient
AI	Artificial Intelligence
CNN	Convolutional Neural Network
CV	Cross-Validation
C-index	Concordance Index
CT	Computed Tomography
DOF	Deep Orthogonal Fusion
DCA	Decision Curve Analysis
EGFR	Epidermal Growth Factor Receptor
HCC	Hepatocellular Carcinoma
ICC	Intrahepatic Cholangiocarcinoma
ICI	Immune Checkpoint Inhibitor
LSTM	Long Short-Term Memory
MIL	Multiple Instance Learning
MLP	Multilayer Perceptron
MRI	Magnetic Resonance Imaging
NSCLC	Non–Small Cell Lung Cancer
PDAC	Pancreatic Ductal Adenocarcinoma
PCA	Principal Component Analysis
PRISMA	Preferred Reporting Items for Scoping Reviews and Meta-Analyses
RF	Random Forest
ROC	Receiver Operating Characteristic
SVM	Support Vector Machine
T1C	T1-weighted Contrast-Enhanced MRI
WGS	Whole-Genome Sequencing
WSI	Whole Slide Image
XGBoost	Extreme Gradient Boosting Algorithm
